# Bioinformatics analysis of the potential receptor and therapeutic drugs for Alzheimer’s disease with comorbid Parkinson’s disease

**DOI:** 10.3389/fnagi.2024.1411320

**Published:** 2024-06-04

**Authors:** Xuerong Zhou, Zhifan Liu, Guiqin Bai, Bai Dazhang, Peilin Zhao, Xiaoming Wang, Guohui Jiang

**Affiliations:** ^1^Department of Neurology, Affiliated Hospital of North Sichuan Medical College, Institute of Neurological Diseases, North Sichuan Medical College, Nanchong, China; ^2^Department of Basic Medicine and Forensic Medicine, North Sichuan Medical College, Nanchong, China

**Keywords:** bioinformatics, Alzheimer’s disease, Parkinson’s disease, comorbidity, epidermal growth factor receptor, Semagacestat

## Abstract

**Background:**

Now, there are no sensitive biomarkers for improving Alzheimer’s disease (AD) and comorbid Parkinson’s disease (PD). The aim of the present study was to analyze differentially expressed genes (DEGs) in brain tissue from AD and PD patients via bioinformatics analysis, as well as to explore precise diagnostic and therapeutic targets for AD and comorbid PD.

**Methods:**

GFE122063 and GSE7621 data sets from GEO in NCBI, were used to screen differentially expressed genes (DEGs) for AD and PD, and identify the intersected genes, respectively. Intersected genes were analyzed by Gene Ontology (GO) analysis and Kyoto Encyclopedia of Genes and Genomes (KEGG) analysis. Then, STRING site and Cytoscape were used to construct a protein–protein interaction (PPI) network, CytoNCA algorithm to analyze and evaluate centrality, Mcode plug-in to analyze module, and Cytohubba to screen key genes. Combined GO-KEGG enrichment analysis with Cytoscape algorithm to screen the key gene in AD complicated with PD. Then, the DEGs for AD and PD were imported into the Association Map (CMap) online platform to screen out the top 10 small molecule drugs, and using molecular docking techniques to evaluate the interactions between small molecule drugs and key genes receptors.

**Results:**

In total, 231 upregulated genes and 300 downregulated genes were identified. GO analysis revealed that the DEGs were highly enriched in signal transduction, and KEGG analysis revealed that the DEGs were associated with the MAPK and PI3K-Akt signaling pathways. Epidermal growth factor receptor (EGFR) was identified as a potential receptor gene in AD and comorbid PD. EGFR was upregulated in both AD and PD, and the proteins that interact with EGFR were enriched in the Ras/Raf/MAPK and PI3K/Akt signaling pathways. Semagacestat was identified as a drug with therapeutic potential for treating AD complicated with PD. There was a high binding affinity between semagacestat and EGFR_NTD_, with seven hydrogen bonds and one hydrophobic bond.

**Discussion:**

Semagacestat may improve the health of patients with AD complicated with PD through the regulation of the Ras/Raf/MAPK and PI3K/Akt signaling pathways by EGFR, providing evidence supporting the structural modification of semagacestat to develop a more effective drug for treating AD complicated with PD.

## Graphical abstract

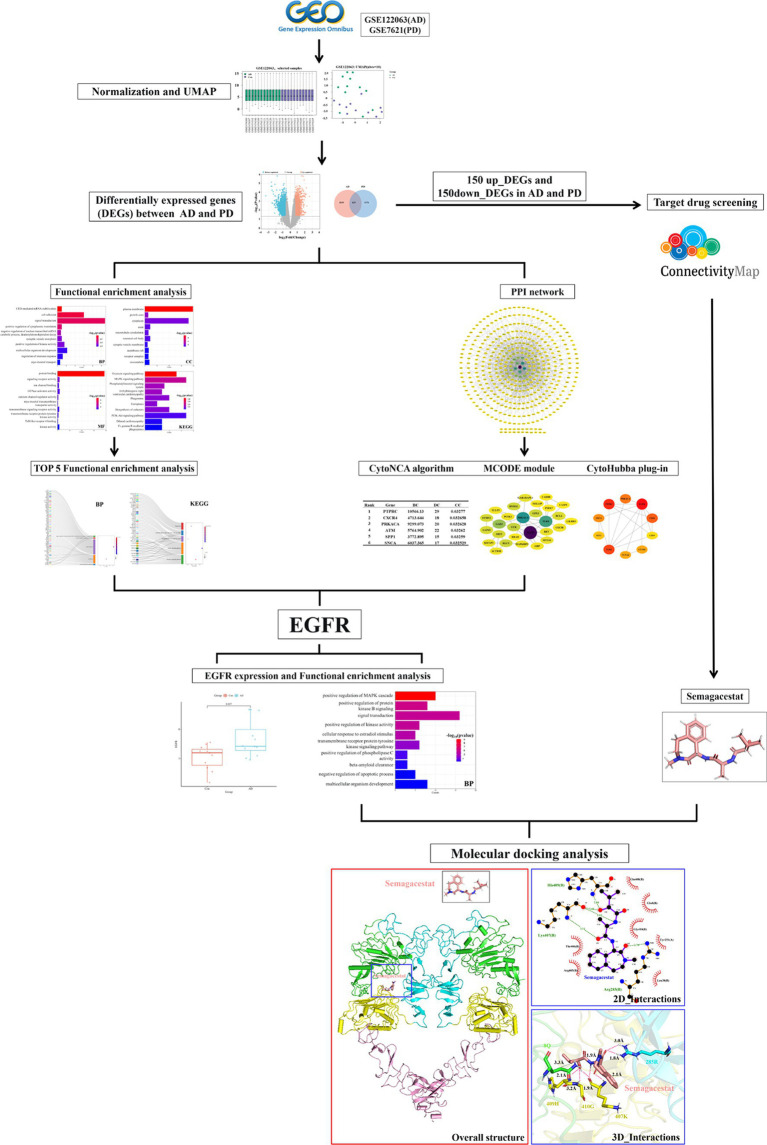

## Highlights

- Epidermal growth factor receptor (EGFR) was identified as a potential crucial receptor gene in Alzheimer’s disease (AD) and Parkinson’s disease (PD) comorbidity via bioinformatics analysis.
- Semagacestat was identified as a drug candidate for AD combined with PD through gene set enrichment analysis.
- Molecular docking revealed that there is a high binding affinity between semagacestat and EGFR_._

## 1 Introduction

Alzheimer’s disease (AD) is a neurodegenerative disease characterized by progressive memory and cognitive impairment, as well as mental and behavioral abnormalities (Masters et al., 2015). The clinical features of AD mainly include progressive memory, cognitive, and emotional dysfunction, as well as behavioral abnormalities. The typical pathological manifestations of AD include amyloid deposition (amyloid β-protein, Aβ), neurofibrillary tangle (NFT) formation, neuronal number reduction, axonal abnormalities, synaptic abnormalities, and granular vacuolar degeneration (Masters et al., 2015). AD can be divided into familial AD and sporadic AD. Familial AD is hereditary and accounts for approximately 4–8% of AD cases. Familial AD is caused mainly by mutations in the amyloid precursor protein (APP), presenilin 1 (PSEN1), and presenilin 2 (PSEN2) genes (Hoogmartens et al., 2021), which lead to Aβ_1-42_/Aβ_1-40_ overexpression and deposition in brain tissue. The etiology of sporadic AD is currently unclear, but the following hypotheses have been proposed: Aβ toxicity hypothesis, tau protein phosphorylation hypothesis, oxidative stress hypothesis, neuroinflammation hypothesis, mitochondrial dysfunction hypothesis, cholinergic damage hypothesis, and apolipoprotein E (ApoE) hypothesis (Zetterberg and Mattsson, 2014).

Parkinson’s disease (PD) is another common neurodegenerative disease. Diffusion weighted magnetic resonance imaging (dw-MRI), particularly diffusion tensor imaging (DTI), has confirmed a reduction in fractional anisotropy (FA) and an increase in mean diffusivity in the substantia nigra in PD patients (Monnot et al., 2017). In addition to a large reduction in the striatal binding ratio (SBR) and dopamine transporter (DAT) levels, cortical and subcortical VMAT2 neurons are reduced in PD (Zhou, 2021a). There are currently several hypotheses regarding the pathogenesis of PD as follows: oxidative stress hypothesis, neuroinflammation hypothesis, neurotoxicity hypothesis, and mitochondrial dysfunction hypothesis (Morris et al., 2024). The main clinical manifestations of PD include motor and nonmotor symptoms. The main motor symptoms include increased overall muscle tension, muscle rigidity, reduced voluntary movement, slow movements, and stiff facial expressions, and these symptoms are often accompanied by static tremors (Magrinelli et al., 2016); PD patients may also experience nonmotor symptoms, such as low mood, anxiety, sleep disorders, cognitive impairment, and fatigue.

It has been reported that the prevalence rate of comorbid mild cognitive impairment (MCI) in PD patients is approximately 20–30%, while for long-term PD with dementia (PDD) patients, the rate can reach 80% (Zhou, 2021a). Additionally, the annual progression rate of PD-MCI to PDD incidence is approximately 11%, and the rate of PDD conversion is greater than 90% after a long time (>15 years) (Zhou, 2021b). Moreover, approximately 50% of AD patients have Lewy bodies, Aβ, and tau lesions in the brain (Azar et al., 2020). With the development of AD, alterations in the activity of enzymes associated with various neurotransmitters (DA, Ach, and GABA) may occur, resulting in imbalances among different neurotransmitter systems, impaired motor function, and even the appearance of PD symptoms, such as motor disorders (Wang et al., 2021). At present, there is no effective option for the treatment of patients with AD and comorbid PD.

Epithelial growth factor receptor (EGFR) is a multifunctional glycoprotein with tyrosine kinase activity that belongs to the ErbB receptor family, which includes HER1 (erbB1 and EGFR), HER2 (erbB2 and NEU), HER3 (erbB3), and HER4 (erbB4) (Sabbah et al., 2020). EGFR, which is widely distributed on the cell membranes of various tissues in the human body, is a receptor for epithelial growth factor (EGF) that promotes cell proliferation and signal transduction. Activated EGFR can alleviate glutamate-induced neurotoxicity and thus exert a neuroprotective effect (Abe and Saito, 1992). EGFR are known to improve both behavioral and pathologic hallmarks of neurodegenerative diseases via autophagy induction and rescuing reactive astrocyte (Romano and Bucci, 2020; Tavassoly et al., 2020). Extensive Aβ_1–42_ production and tau phosphorylation form because of overexpressed EGFR leading to sustained phosphorylation of the downstream signaling axis (Jayaswamy et al., 2023). In transgenic Drosophila and transgenic mouse models, EGFR is the preferred target for treating Aβ-induced memory loss, suggesting that EGFR plays a key role in maintaining neuronal structure and function (Sibilia and Wagner, 1995). In addition, the EGFR signaling pathway and its related genes play important roles in the death of DA neurons (Kim et al., 2017; Jin et al., 2020). EGFR aggregates to form dimers when activated by ligands, subsequently regulating downstream signaling pathways through TK activation and phosphorylation of tyrosine residues in the CTD. According to KEGG pathway analysis, EGFR-mediated regulation of AD and comorbid PD may involve the RAS/RAF/MAPK pathway and PI3K/Akt pathway (Gao et al., 2023; Nowell et al., 2023). In the RAS/RAF/MAPK pathway, when EGFR activates Ras, it promotes the conversion of plasma membrane RAS-GDP to the RAS-GTP active form, which activates the Raf protein kinase. Activated Raf activates ERK kinase, which amplifies signals to induce the ERK cascade through positive feedback, subsequently promoting the proliferation of nerve cells and axonal growth (Zhang et al., 2021; Chun et al., 2022). The PI3K/Akt signaling pathway regulates glucose homeostasis and energy metabolism in the brain through the insulin signaling pathway, thereby affecting synaptic function, learning, and working memory (De Felice et al., 2022). mTOR is the initiator protein of autophagy. The PI3K/Akt signaling pathway affects the autophagic function of nerve cells through mTOR, mediating the degradation of pathological proteins, the accumulation of neurocytotoxic substances, neuronal survival, axonal regeneration, and synaptic plasticity (Liu et al., 2017; Yang et al., 2023). The PI3K/Akt signaling pathway regulates the activity of GSK-3β, resulting in the hyperphosphorylation of pathological proteins in neurodegenerative diseases (L'Episcopo et al., 2016).

The structure of EGFR is divided into an N-terminal domain (NTD), a single transmembrane domain (TM), and a C-terminal intracellular domain. The N-terminus of EGFR (EGFR_NTD_), a ligand-binding domain that receives external signals, is divided into the following four subregions: region I is responsible for binding ligands; region II interacts with ligands through conserved amino acid residues (Sabbah et al., 2020); and regions III and IV are involved in the formation of intramolecular disulfide bonds. The transmembrane (TM) domain is a transmembrane hydrophobic region, consisting of 23 amino acid residues, which forms an α-helical structure and anchors EGFR to the cell membrane. The C-terminal intracellular carboxy-terminal region, which has a conserved tyrosine kinase core, can be further divided into the near-membrane (JM) region, tyrosine kinase (TK) region, and C-terminal domain (CTD) region. The JM region regulates the dimerization of EGFR after it binds to ligands, which is followed by ATP binding and TK activation; ultimately, the CTD undergoes autophosphorylation and regulates intracellular signal transduction pathways (Shao and Zhu, 2019).

In the present study, changes in the expression profiles of genes in brain tissue samples from AD and PD patients were analyzed. DEGs in both AD and PD patients were identified. Gene Ontology (GO) term and Kyoto Encyclopedia of Genes and Genomes (KEGG) pathway enrichment analyses were performed, and a protein–protein interaction network (Genomes) was constructed. The MCODE plugin, CytoHubba plug-in, and CytoNCA algorithms were used to identify key genes related to AD and comorbid PD (Nayan et al., 2023; Zhou et al., 2023). A connectivity map (CMap) was generated to identify the optimal drug for treating AD and comorbid PD. Finally, molecular docking was performed with AutoDock to evaluate the interactions between the key proteins involved in AD and comorbid PD, as well as between the key proteins and optimal drugs, providing a theoretical basis for improving the quality of life of patients with AD and comorbid PD.

## 2 Materials and methods

### 2.1 Data sources, quality and variance analysis

The GSE122063 (AD) and GSE7621 (PD) gene expression datasets were obtained from the GEO database. The GSE122063 dataset comprises gene expression data for the frontal lobe of Alzheimer’s disease patients. The GSE7621 dataset comprises gene expression data from the substantia nigra tissues of Parkinson’s disease patients. The differences in gene expression profiles between AD patient and PD patient samples were analyzed using the GEO2R online platform (http://ncbi.nlm.nih.gov/geo/geo2r). Uniform manifold approximation and projection (UMAP) analysis was performed to assess homogeneity, repeatability, and differences between the groups. Samples selected with a median that is basically on the same horizontal line for subsequent analysis (Nayan et al., 2023; Zhou et al., 2023).

### 2.2 Differential expression gene

After preliminary analysis, DEGs were obtained with the following screening criteria: *p* < 0.05 and |LogFC| > 0.585. A logFC >0.585 indicated upregulation (upregulated DEGs), and a logFC < −0.585 indicated downregulation (downregulated DEGs). The following graphing website was used to generate a volcano plot of the DEGs: (http://www.bioinformatics.com.cn). The overlapping DEGs were identified using a Venn diagram, and a heatmap was generated.

### 2.3 Analyses of the DEGs by enrichment analyses

GO term and KEGG pathway enrichment analyses of the overlapping DEGs were performed with the DAVID tool (https://david.ncifcrf.gov/summary.jsp). GO analysis is a common method used to study gene function and includes biological process (BP), cellular component (CC), and molecular function (MF) information associated with certain genes. KEGG pathway analysis, which identifies specific pathways associated with DEGs, uses a large amount of information about the genome, disease, biological pathways, and system function to help identify metabolic pathways that are significantly altered in disease states.

### 2.4 Screening key genes by the PPI network

The interactions of the proteins encoded by the overlapping DEGs were analyzed using the STRING online platform (https://string-db.org/). The minimum interaction threshold was set to medium confidence (0.4), and the results were imported into Cytoscape 3.9.1 to construct a protein–protein interaction (PPI) diagram. In the PPI network diagram, the lines between nodes indicate direct interactions. Nodes with a higher number of connected lines have more important roles in the PPI network. The MCODE plug-in was used to analyze the most closely related gene clusters in the PPI network. The CytoHubba module (MCC, MNC, Degree, EPC, Betweenness, and Closeness) was used to select the top 10 genes with the highest connectivity in the PPI network. The CytoNCA algorithm was used to calculate the betweenness centrality (BC), degree centrality (DC), and closeness centrality (CC) in the PPI network, as well as to determine the top 10 targets according to the BC, CC, and DC values. By combining the results of various algorithms, the key receptor genes involved in AD and comorbid PD were identified.

### 2.5 Expression and functional enrichment analysis of receptor proteins

The gene expression of key receptors in the AD and PD datasets was analyzed, and a box plot was generated using the Weisheng platform. GO_BP enrichment and KEGG pathway analyses of the proteins that directly interact with the key receptor genes were performed using the DAVID database.

### 2.6 Target drug screening

The Connectivity Map platform (CMap, https://clue.io/) was used to identify drug candidates for AD and PD. The CMap platform was used to predict, analyze, and identify drug candidates for particular diseases through gene set enrichment analysis (GSEA) to evaluate the overlap between the imported DEGs and the genes whose expression was altered by treatment with small molecule drugs from a database. The upregulated and downregulated genes whose encoded proteins are localized to the plasma membrane in AD and PD patients were uploaded to the CMap platform. Small molecules with low scores were selected as candidate drugs for alleviating AD and comorbid PD.

### 2.7 Evaluation of the interaction between receptor proteins and target drugs

The drug structures were obtained from PubChem (https://pubchem.ncbi.nlm.nih.gov), a small molecule database, and the protein structures were obtained from the PDB database (https://www.rcsb.org), a protein structure database. The small molecule and receptor protein structures were imported into AutoDockTools 4.2.6 software, and after hydrogenation, charge distribution, and other processing, molecular docking was performed to obtain the binding energy (kcal/mol) between the drugs and key targets. The interactions between drugs and receptors were analyzed by LigPlot^+^ software and the PILP website (https://plip-tool.biotec.tu-dresden.de/plip-web/plip/index), and they were visualized by PyMOL software.

## 3 Results

### 3.1 Quality and variance of AD and PD datasets

**Figure 1:**
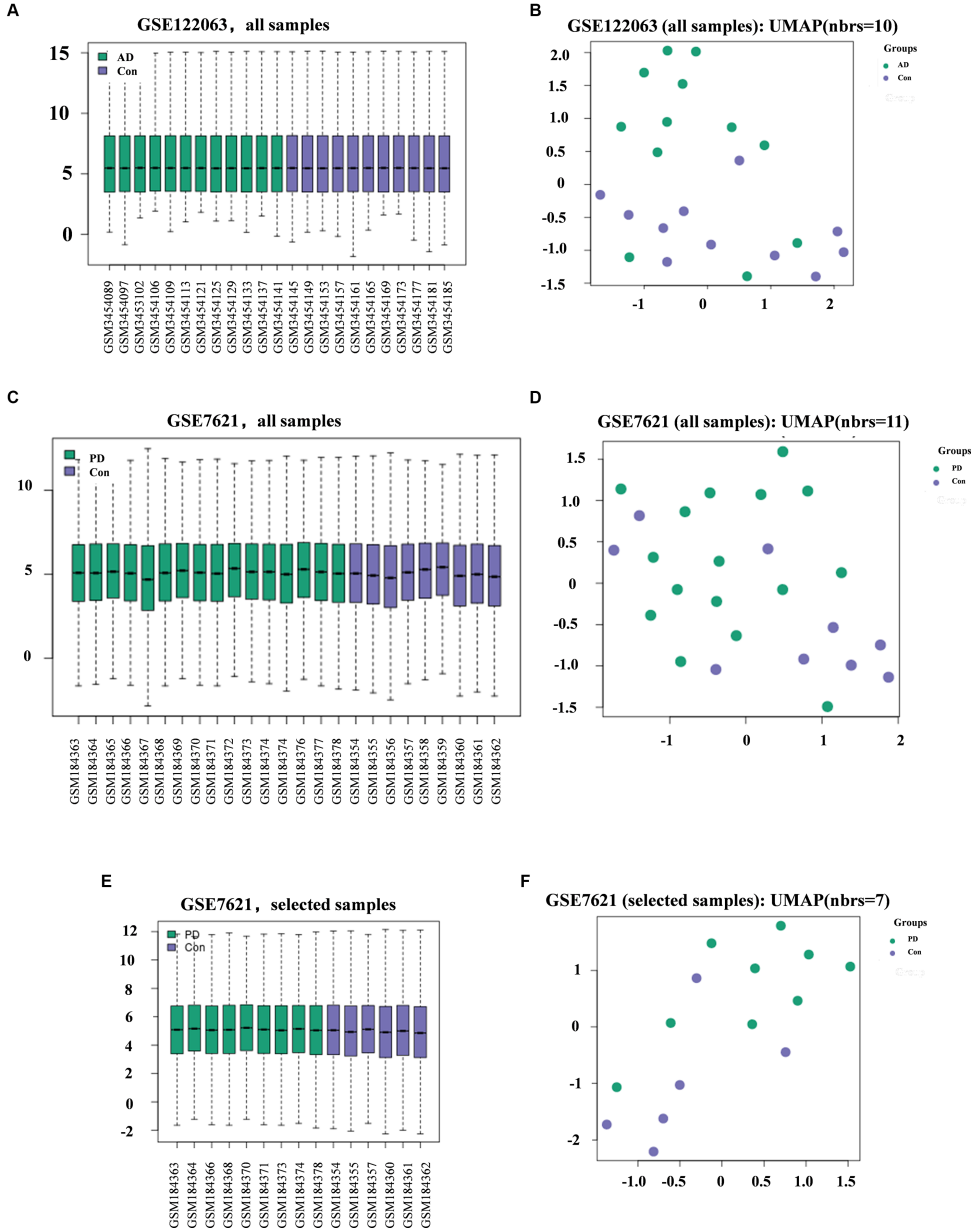
Diagram showing the quality of the data from the AD and PD datasets and the differences between groups. Quality **(A)** and difference **(B)** analysis of data in the AD datasets. Quality **(C)** and difference **(D)** analysis of data for all samples **(C-D)** or selected samples **(E-F)** in the PD datasets.

The GEO2R platform was used for standardization and UMAP analysis of the samples included in the AD and PD datasets. In the AD dataset, all the samples from were selected, including 11 healthy controls and 12 AD patients. In the PD dataset, the selective study included 6 healthy controls and 9 PD patients (Table 1; Figures 1C–F). The results showed that the median values of all the samples were horizontal (Figures 1A,B), and the distance between the samples from the two groups was relatively large (Figures 1C,D), indicating a good degree of normalization between the samples and small differences between the datasets.

**Table 1:** Accession numbers of samples from the GSE122063 (AD) and GSE7621 (PD) datasets.

	GSE122063 (AD)	GSE7621 (PD)
Accession numbers	GSM3454089, GSM3454097, GSM3453102, GSM3454106, GSM3454109, GSM3454113, GSM3454121, GSM3454125, GSM3454129, GSM3454133, GSM3454137, GSM3454141, GSM3454145, GSM3454149, GSM3454153, GSM3454157, GSM3454161, GSM3454165, GSM3454169, GSM3454173, GSM3454177, GSM3454181, GSM3454185	GSM184354, GSM184355, GSM184357, GSM184360, GSM184361, GSM184362, GSM184363, GSM184364, GSM184366, GSM184368, GSM184370, GSM184371, GSM184373, GSM184374, GSM184378

### 3.2 Identified DEGs of the AD and PD datasets

**Figure 2:**
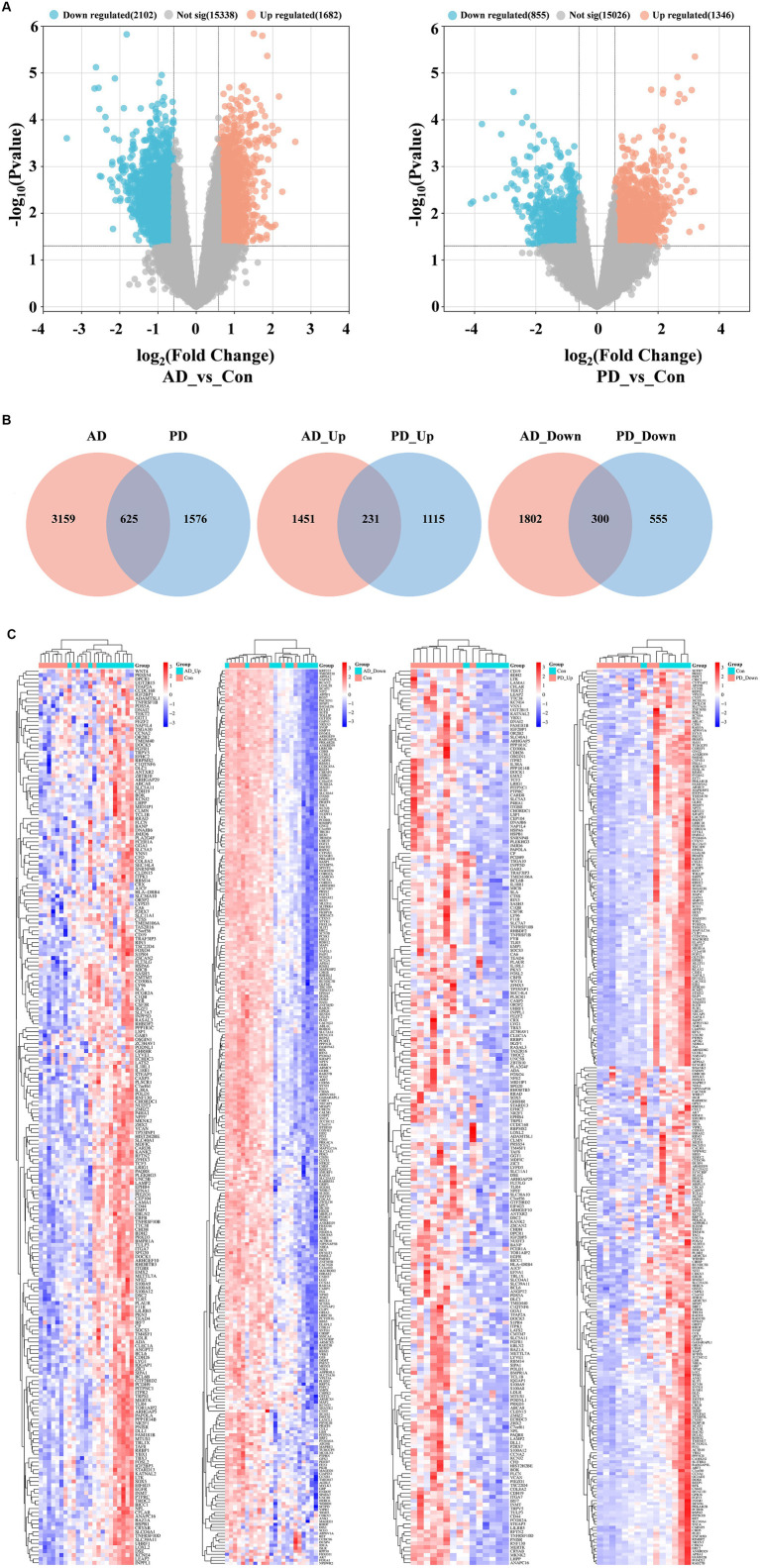
Analysis of DEGs in the AD and PD datasets. **(A)** Volcano plot. **(B)** Venn diagram. **(C)** Complex heat map.

Analysis of the gene expression data in the AD and PD datasets revealed 3,784 DEGs and 2,201 DEGs, respectively (Figure 2A), and 625 DEGs were obtained after intersecting the two groups of DEGs. A total of 531 genes were differentially expressed in both the AD and PD datasets, with 231 upregulated DEGs and 300 downregulated DEGs (Figure 2B). The overlapping DEGs between the two datasets are shown in a heatmap (Figure 2C).

### 3.3 Enrichment analysis of DEGs

**Figure 3:**
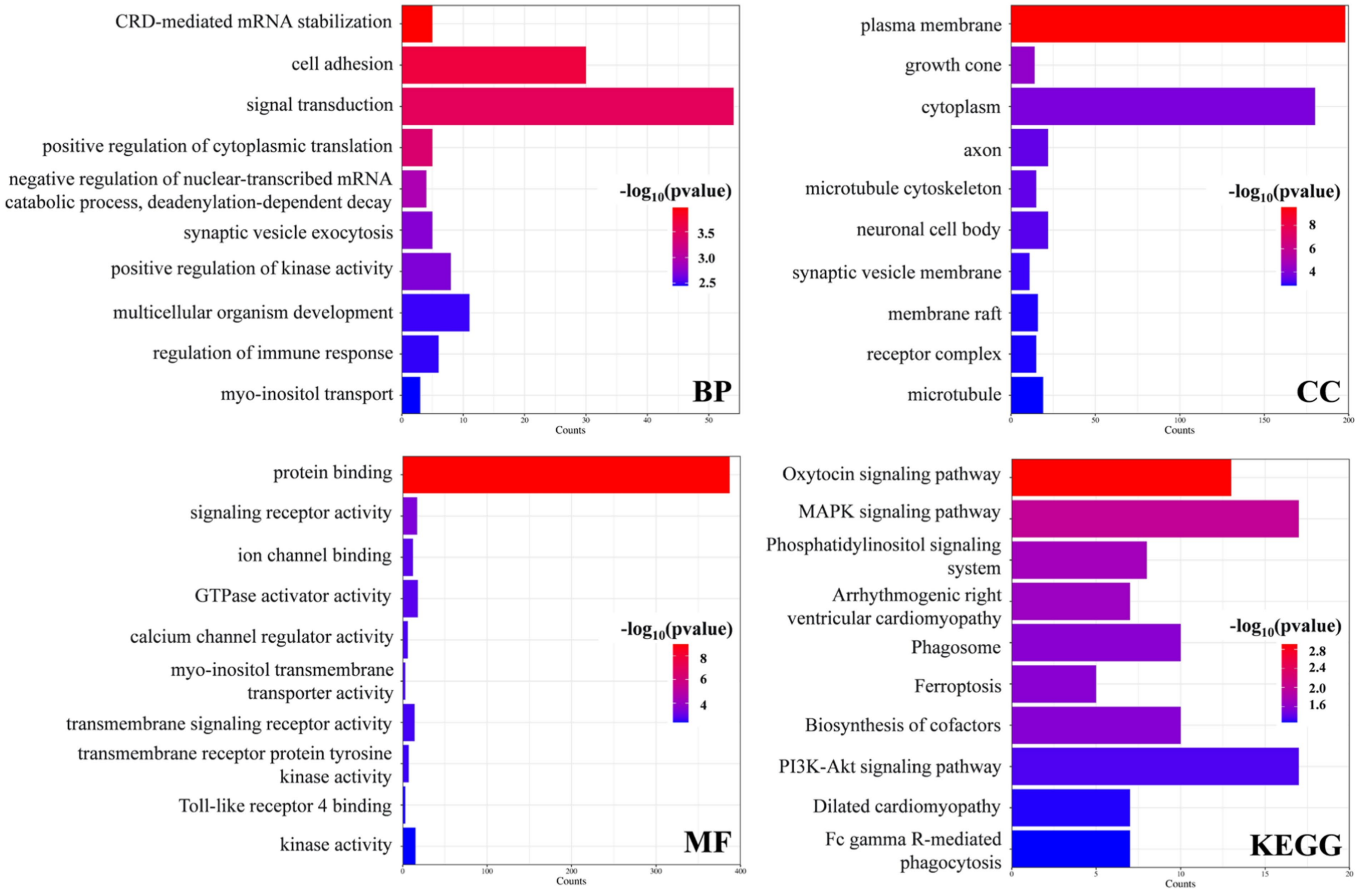
Enrichment analysis of the DEGs in the AD and PD datasets.

**Figure 4:**
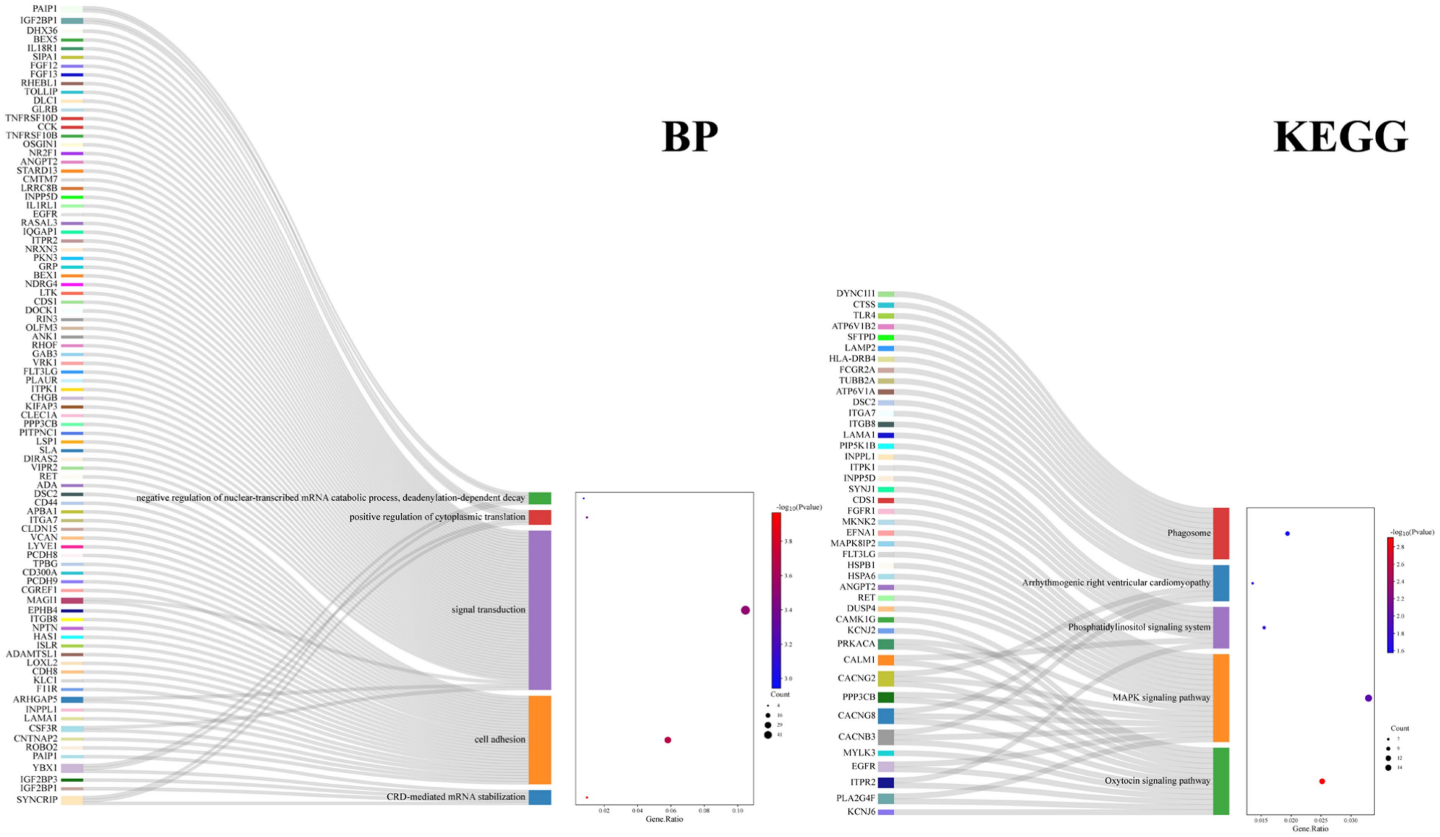
Enrichment analysis of the DEGs in BP and KEGG for top 5.

GO term and KEGG pathway enrichment analyses of the overlapping DEGs between the AD and PD datasets were performed (Figure 3). The main enriched BP terms included signal transduction, cell adhesion, development of multicellular organisms, positive regulation of kinase activity, and regulation of immune response. The main enriched CC terms were plasma membrane, cytoplasm, axon, neuron cell bodies, and microtubule. The main enriched MF terms were protein binding, GTPase activator activity, signal receptor activity, kinase activity, transmembrane signal receptor activity, and other molecular functions. The enriched KEGG pathways were the MAPK signaling pathway, the PI3K/Akt signaling pathway, the oxytocin signaling pathway, phagosome and cofactor biosynthesis, and other signaling pathways. Further analysis of the GO and KEGG pathway enrichment analyses identified the following genes in all four enrichment analyses: RET, ANGPT2, FLT3LG, EGFR, PPP3CB, FGFR1, MAGI1, CSF3R, ITGB8, ITGA7, and FCGR2A. In addition, the encoded proteins of these genes are localized on the cell membrane (Figure 4).

### 3.4 Identified key gene of the DEGs

**Figure 5:**
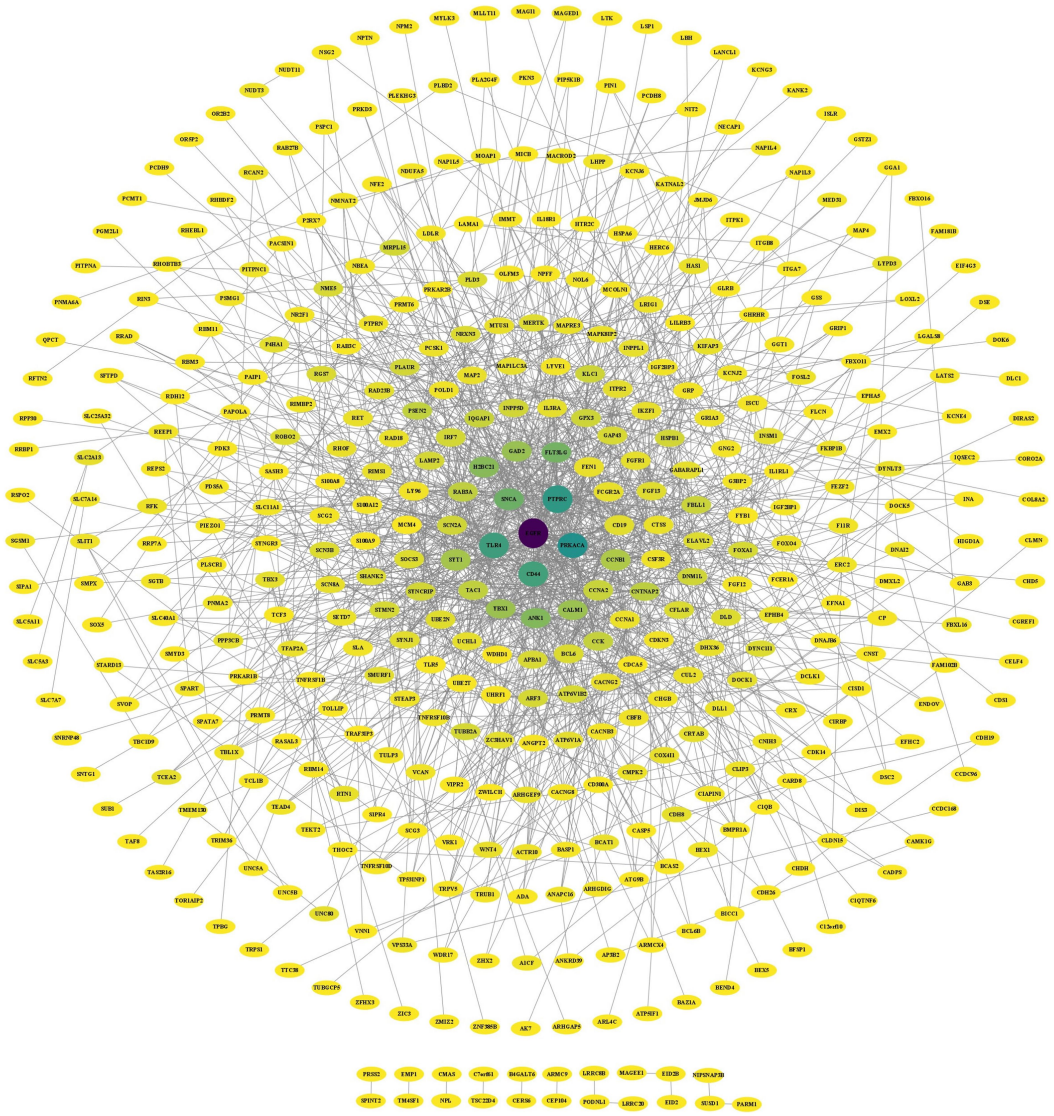
PPI network of the DEGs in the AD and PD datasets.

The results of the STRING analysis were analyzed with Cytoscape software, which obtained 1,191 direct interactions among 434 receptors (Figure 5). BC indicates the extent to which a node acts as a hub in the network, and DC represents the number of connections between nodes. Moreover, CC represents the closeness of the connections between nodes. Larger DC and BC values of a node indicate stronger interactions with other nodes and more downstream nodes it regulates (Yu et al., 2007). In the PPI network, darker colors represent higher BC values, and larger DC values represent larger nodes. After analysis of the PPI network using the BC, CC, and DC algorithms of CytoNCA, the 7 genes whose BC, CC and DC values were among the 10 highest were retained (Table 2).

**Table 2:** Ranking of the DEGs in the AD and PD datasets according to the CytoNCA algorithm.

Rank	Node	DC	BC	CC
1	EGFR	54	36454.562	0.042888273
2	PTPRC	46	16933.275	0.042530205
3	TLR4	39	15698.312	0.04254692
4	CD44	37	15279.223	0.042505153
5	PRKACA	37	18139.5	0.042442657
6	SNCA	25	11824.844	0.042355474
7	GAD2	21	8256.892	0.04213702

**Figure 6:**
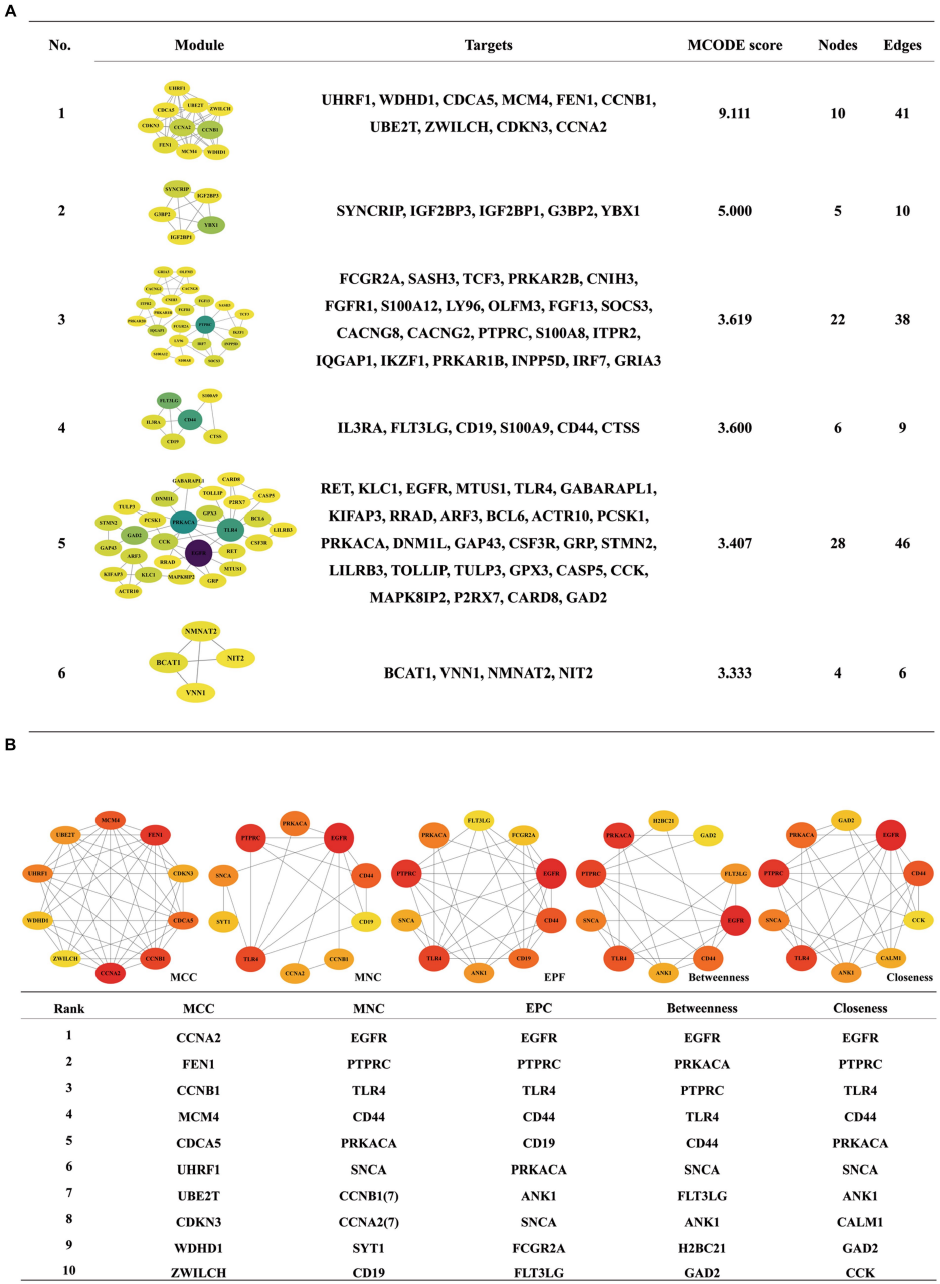
Analysis of the DEGs in the PPI network by the MCODE **(A)** and CytoHubba **(B)** algorithms.

The PPI network diagram was constructed using the clustering function of the MCODE module. Six modules with scores greater than 3 were retained, with scores of 9.111, 5.000, 3.619, 3.600, 3.407, and 3.333 (Figure 6A). The top 10 genes with the highest connectivity were identified by the MCC, MNC, EPC, closeness, and betweenness algorithms of the CytoHubba plug-in (Figure 6B). Combining the GO term and KEGG pathway enrichment analyses with the results obtained via the MCODE plugin, CytoHubba plug-in, and CytoNCA algorithm identified the epidermal growth factor receptor (EGFR) as the most likely critical receptor gene in AD and comorbid PD.

### 3.5 Functional enrichment analysis of EGFR

**Figure 7:**
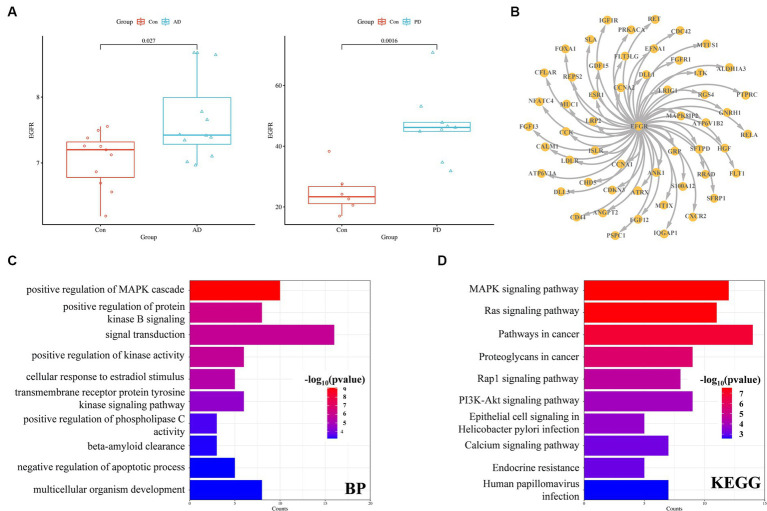
EGFR expression and interaction partners. **(A)** EGFR expression in the AD and PD datasets. **(B)** Proteins that interact with EGFR **(C)** GO enrichment **(C)** and KEGG pathway **(D)** analysis of proteins that interact with EGFR.

EGFR expression was significantly increased in both the AD and PD datasets (Figure 7A). Among the overlapping DEGs in the AD and PD datasets, there were 54 encoded proteins that directly interacted with EGFR (Figure 7B). The genes associated with these proteins were enriched mainly in the signal transduction biological process and cancer pathways, including the Ras/Raf/MAPK signaling pathway and the PI3K/Akt signaling pathway (Figures 7C,D).

### 3.6 Identified target drug

CMap was used to identify drug candidates for the treatment of AD and PD. If a small molecule had a negative score, it suggested that it had potential efficacy, and greater negative scores indicated greater efficacy. Among the selected drug candidates, semagacestat (BRD-K65592642) had the most negative score between AD and PD patients (Table 3).

**Table 3:** Identification of drug candidates for the treatment of AD and PD with CMap.

Rank	AD	Score	PD	Score
1	BRD-K65592642	−2.0357	BRD-K65592642	−1.8737
2	Erastin	−1.9666	Proadifen	−1.6172
3	Palovarotene	−1.9492	Risperidone	−1.5911
4	Picotamide	−1.7779	CL-218872	−1.5573
5	Diethylcarbamazine	−1.7757	SB-939	−1.5483
6	BRD-K41170226	−1.7603	BW-723C86	−1.5453
7	CP-724714	−1.758	SNS-314	−1.5449
8	Simeprevir	−1.7559	BMS-536924	−1.525
9	Lofexidine	−1.7511	Ellagic acid	−1.5249
10	Bitopertin	−1.7487	Verapamil	−1.524

### 3.7 Predicted interactions between EGFR and semagacestat

**Figure 8:**
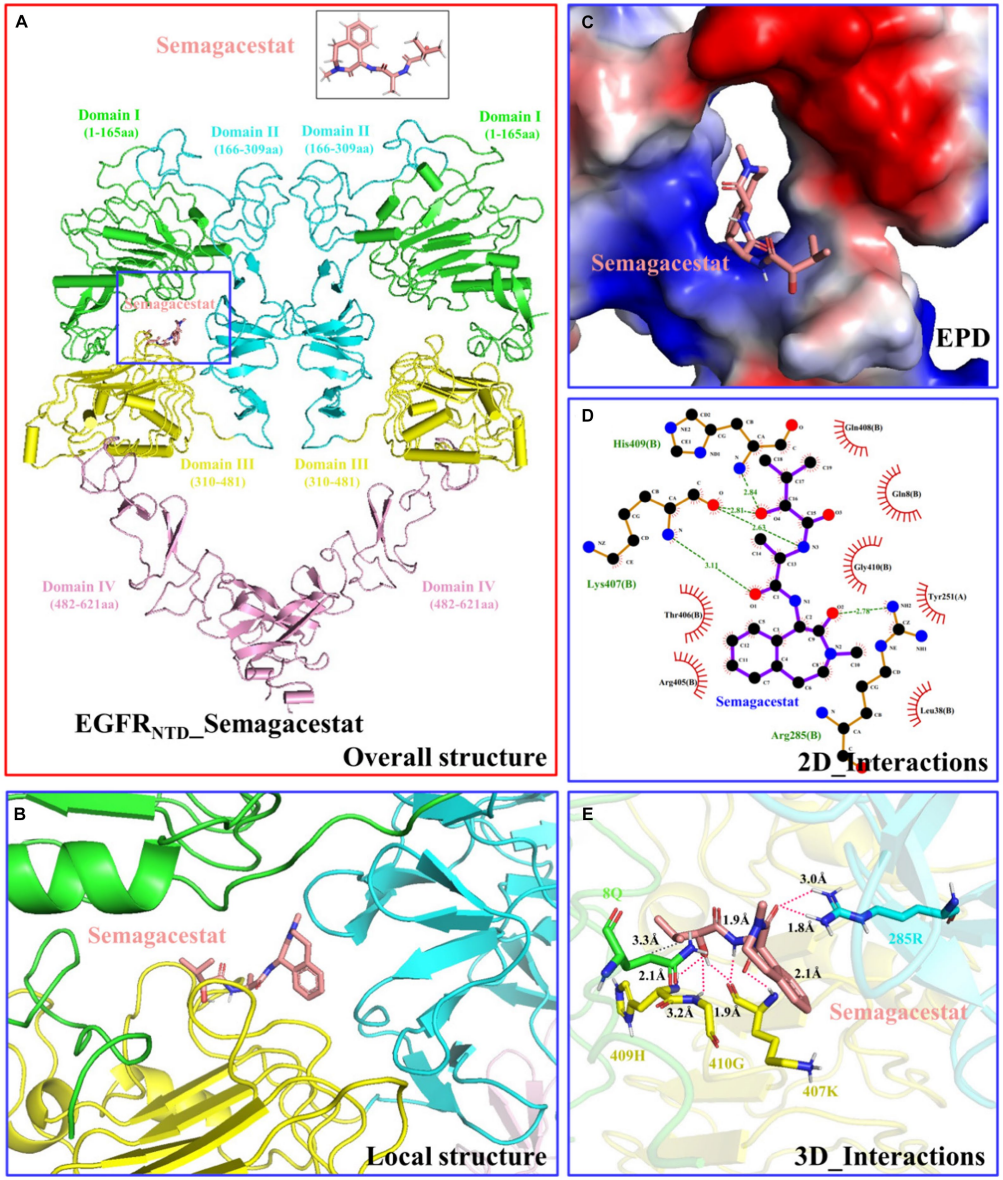
Structure and interaction of the EGFR_NTD__semagacestat complex. **(A)** Overall structure. **(B)** Local structure. **(C)** Electrostatic potential diagram. **(D)** 2D interactions. **(E)** 3D interactions. This section may be divided by subheadings. It should provide a concise and precise description of the experimental results, their interpretation, as well as the experimental conclusions that can be drawn.

The structure of the EGFR N-terminus (EGFR_NTD_, PDB ID: 7SZ7) was obtained from the PDB database, and the structure of semagacestat was obtained from the PubChem database. The molecular docking results obtained from AutoDock are shown in Figure 8 (only structures with a low complex potential energy are shown). The binding energy between semagacestat and EGFR_NTD_ was −7.8 k/mol. Semagacestat was embedded in a cavity formed by regions I, II, and III of EGFR_NTD_ (Figures 8A,B). The electrostatic potential diagram (EPD) of the EGFR_NTD__semagacestat complex indicated positively charged amino acids around semagacestat (Figure 8C), and semagacestat formed seven hydrogen bonds and one hydrophobic bond (8Q) with EGFR_NTD_ (285R, 407 K, 409H, and 410G) (Figures 8D,E).

## 4 Discussion

AD and PD are the most common neurodegenerative diseases in humans. AD is a degenerative disease of the central nervous system whose main pathological feature is the formation of Aβ plaques and neurofibrillary tangles in the brain, which affect cognitive function, memory, ability to perform life activities, emotions, and personality. PD involves the loss of dopaminergic neurons in the substantia nigra pars compacta and the formation of Lewy bodies in dopaminergic neurons and Lewy neurites in multiple brain areas, resulting in motor dysfunction. In the advanced stage of AD, pathological characteristics associated with both AD and PD may be present in the brain tissue of patients and have dual impacts on physical health and quality of life.

In the present study, the brain tissue sample gene expression data from the GSE120063 (AD) and GSE7621 (PD) datasets were analyzed, and UMAP analysis revealed little difference between the samples from the AD and PD groups. DEGs in the datasets were identified according to the following criteria: *p* < 0.05 and |LogFC| > 0.585. A total of 3,784 DEGs and 2,201 DEGs were identified in the AD and PD datasets, respectively. There were 625 overlapping DEGs, with 231 upregulated DEGs and 300 downregulated DEGs. To assess the relationships among the genes, the PPI network was analyzed with the CytoNCA algorithm, MCODE module, and CytoHubba plug-in, which suggested that EGFR may be a potential receptor in AD and comorbid PD. EGFR expression was significantly increased in the AD and PD datasets. Enrichment analysis revealed that 54 proteins that directly interact with EGFR were enriched in the signal transduction biological process and in cancer pathways, such as the MAPK signaling pathway.

Semagacestat (BRD-K65592642) was among the small molecule drug candidates for AD and PD identified by CMap. Semagacestat is the most widely studied gamma-secretase inhibitor, and it blocks hybrid β sheet formation between substrates and presenilin 1 to inhibit substrate cleavage (Tagami et al., 2017; Hur, 2022). Stable isotope labeling kinetics have shown that semagacestat decreases the levels of Aβ_1-38_, Aβ_1-40_, and Aβ_1-42_ in cerebrospinal fluid but increases the levels of Aβ_1-15_ and/or Aβ_1-16_ (Hölttä et al., 2016); however, no decrease in the level of Aβ_1-42_ or Aβ_1-40_ in cerebrospinal fluid has been detected in phase III trials. Moreover, the use of semagacestat was stopped in August 2010 due to adverse reactions in phase III trials (Lleó et al., 2015). The failure of the clinical evaluation of semagacestat may be caused by multiple factors, one of which is thought to be related to its half-life (2–3 h). Due to its short half-life, semagacestat must reach a high concentration to inhibit Aβ production, which can also inhibit the cleavage of Notch and other substrates, resulting in side effects (De Strooper, 2014; Yang et al., 2021).

According to molecular docking analysis of EGF and semagacestat, the binding energy between semagacestat and EGFR_NTD_ was −7.8 k/mol, which is less than −5 k/mol, indicating a high binding affinity between semagacestat and EGFR_NTD_. Semagacestat was found to be embedded in the cavity formed by the EGFR_NTD_ domain I, II, and III regions, which agreed with a previous study (Sabbah et al., 2020). Seven hydrogen bonds and one hydrophobic bond formed between semagacestat and EGFR_NTD_, and these bonds are key for the effect of semagacestat on the activity of EGFR_NTD_ (Zhou et al., 2022; Zheng et al., 2023). Although semamacestat can inhibit the activity of gamma-secretase and potentially improve the pathology of AD combined with PD through EGFR, it can also exacerbate side effects by inhibiting the cleavage of Notch. Therefore, the structure of sematacestat can be optimized based on the structure of the EGFR_NTD__semagacestat complex to improve the substrate-selective design. Despite these many unresolved questions, the present study revealed the molecular basis for the recognition and mechanism of action of semagacestat and EGFR, suggesting that semamacestat may improve the pathology of AD and comorbid PD through EGFR, which may ultimately lead to potent therapeutics targeting AD and comorbid PD.

In summary, the present study utilized bioinformatics methods to analyze gene expression in AD and PD patients. According to the enrichment analysis of the DEGs in AD and PD patients, as well as the PPI network map analysis, EGFR may be a key receptor in patients with AD and comorbid PD. According to the GSEA algorithm in CMap, semagacestat is a potential small molecule drug for the treatment of AD and comorbid PD. Molecular docking analysis revealed information about the interaction and binding between semagacestat and EGFR, which may provide a rationale for further analysis of EGFR inhibitors designed on the basis of the semagacestat structure. Thus, the present study provides a novel strategy for the development of drugs for the treatment of AD and comorbid PD.

## 5 Conclusion

The present results suggested that EGFR may be a key receptor in AD and comorbid PD. EGFR may mediate the development of AD and PD through cancer-related pathways, such as the Ras/Raf/MAPK and PI3K/Akt pathways. Semagacestat (BRD-K65592642) is a potential candidate drug for treating AD complicated with PD. Molecular docking analysis of the interaction between semagacestat and EGFR suggested that modification of the semagacestat structure may be a novel strategy for the development of more effective drugs for AD and comorbid PD.

## Data availability statement

Publicly available datasets were analyzed in this study. These data may be found in the GSE122063 (https://www.ncbi.nlm.nih.gov/geo/query/acc.cgi?acc=GSE122063) and GSE7621 (https://www.ncbi.nlm.nih.gov/geo/query/acc.cgi?acc=GSE7621) datasets.

## Ethics statement

Ethical approval was not required for the studies involving humans because GEO belong to public databases. The patients involved in the database have obtained ethical approval. Users can download relevant data for free for research and publish relevant articles. Our study is based on open source data, so there are no ethical issues and other conflicts of interest. The studies were conducted in accordance with the local legislation and institutional requirements. The human samples used in this study were acquired from gifted from another research group. Written informed consent to participate in this study was not required from the participants or the participants’ legal guardians/next of kin in accordance with the national legislation and the institutional requirements.

## Author contributions

XZ: Conceptualization, Funding acquisition, Methodology, Project administration, Visualization, Writing – original draft, Writing – review & editing. ZL: Investigation, Methodology, Writing – review & editing. GB: Investigation, Writing – review & editing. DB: Writing – review & editing. PZ: Writing – review & editing. XW: Writing – review & editing. GJ: Conceptualization, Data curation, Project administration, Writing – review & editing.
